# Production of anhydrous hydrogen fluoride from fluorosilicic acid: a review

**DOI:** 10.3389/fchem.2024.1372981

**Published:** 2024-02-27

**Authors:** Huachun Yang, Shijiang Li, Hehua Yu, Haixia Liu, Kai Sun, Xiaolan Chen

**Affiliations:** ^1^ Do-Fluoride New Materials Co., Ltd., Jiaozuo, China; ^2^ College of Chemistry, Zhengzhou University, Zhengzhou, China

**Keywords:** anhydrous hydrogen fluoride, fluorosilicic acid, fluorine chemistry, phosphine chemistry, green chemistry

## Abstract

Anhydrous hydrogen fluoride (AHF), a critical raw material for industries such as aluminum, pharmaceuticals, and petroleum, has traditionally been sourced from fluorite—a non-renewable mineral. The unsustainable reliance on fluorite has catalyzed the search for alternative AHF production methods. A promising substitute is fluorosilicic acid (FSA), a byproduct of the phosphate fertilizer industry previously deemed waste. Transforming fluorosilicic acid into AHF not only yields a valuable resource but also addresses the environmental and economic challenges associated with waste management. The innovative practice of producing AHF from fluorosilicic acid signals a shift towards sustainable chemical production by capitalizing on waste, potentially diminishing reliance on fluorite and reducing the industry’s environmental impact. This review thoroughly dissects the AHF synthesis process from fluorosilicic acid. Despite the acknowledged importance of fluorinated compounds in numerous industrial applications, research on their synthesis from fluorosilicic acid is limited and fragmented. This review seeks to amalgamate this scattered information by closely scrutinizing diverse industrial processing methods. Additionally, it explores the current and future landscape, economic feasibility, and strategies to navigate the obstacles inherent in synthesizing AHF from fluorosilicic acid. It also assesses the environmental impact of these methods, thereby contributing to the dialogue in this emerging field. The primary aim of this manuscript is to foster further research and promote the industrial uptake of this sustainable process. Highlighting the challenges and proposing potential improvements, the review supports the responsible reuse of waste and advocates for advancements in industrial practices.

## 1 Introduction

**FIGURE 1:**
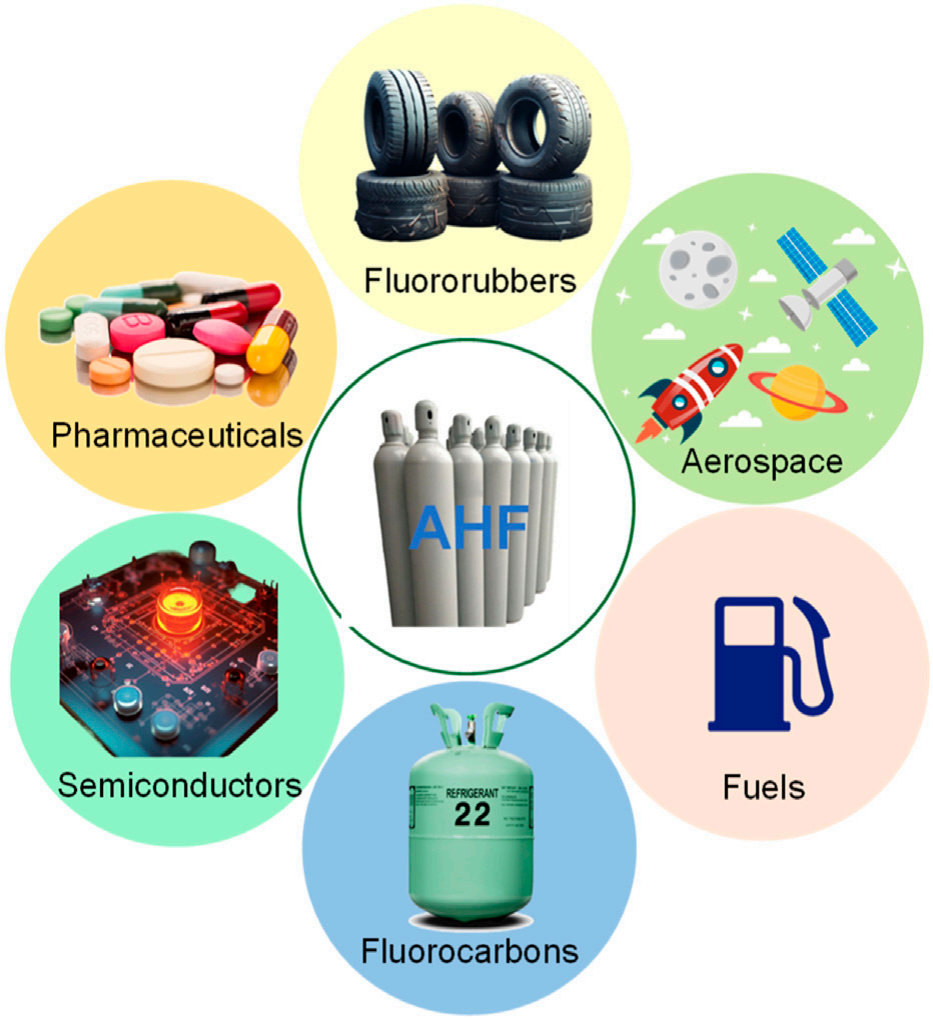
Applications of AHF.

Industrial chemistry has made remarkable advancements, leading to the development of various compounds with versatile applications. Among these, anhydrous hydrogen fluoride (AHF), in particular, has attracted significant attention owing to its expanding utility across diverse industrial sectors (Joshi, 2022; Patel et al., 2023). As a compound consisting of hydrogen and fluorine, AHF is the building block for a wide array of chemical processes and products, serving as an indispensable catalyst and reactant. Its significance spans sectors such as pharmaceuticals, petrochemicals, polymers, and electronics, marking it as a substance of global scientific and industrial interest (Figure 1) (Manteau et al., 2010). One of the primary uses of AHF is in the production of fluorocarbons, which are utilized in refrigeration, air-conditioning systems, and aerosol propellants. These fluorocarbons, particularly hydrochlorofluorocarbons (HCFCs) and hydrofluorocarbons (HFCs), have been critical in replacing chlorofluorocarbons (CFCs) due to their lower ozone-depleting potential (Hyun Cho et al., 1998; Han et al., 2018; Sicard and Baker, 2020). Besides that, it is extensively utilized in the production of fluoroplastics, fluororubbers, and fluorinated pharmaceuticals and pesticides (Gardiner, 2015). In the petrochemical industry, AHF acts as a catalyst in the alkylation process, where it facilitates the combination of olefins with isobutane to produce high-octane components essential for gasoline (de Klerk and de Vaal, 2008). The efficiency of AHF in catalyzing the alkylation reaction makes it a staple in refineries, contributing to the production of cleaner-burning fuels and complying with environmental regulations. Furthermore, the electronic industry heavily relies on AHF for the cleaning and etching of silicon wafers during the manufacturing of semiconductors and integrated circuits. Its precision in etching fine lines allows for the production of smaller and more powerful electronic devices, underlying the technological advancements in the computer and mobile phone industries (Steinert et al., 2005). In the nuclear industry, AHF is used in the enrichment of uranium. Uranium hexafluoride (UF_6_), produced with AHF, is used in gas centrifuge and gaseous diffusion processes to produce enriched uranium, which is a critical step in both civil nuclear power generation and military applications, including the production of weapons-grade materials (Morse and Parry, 1967; Souček et al., 2017). In aerospace, AHF is a component in the synthesis of propellants used in liquid rocket engines (Feng et al., 2009). It helps produce high-energy compounds, such as oxidizers for rocket fuel (Uhlenhake et al., 2022). Additionally, AHF-derived compounds serve as additives to improve the stability and performance of these propellants.

### 1.1 Classical method of AHF production

**FIGURE 2:**
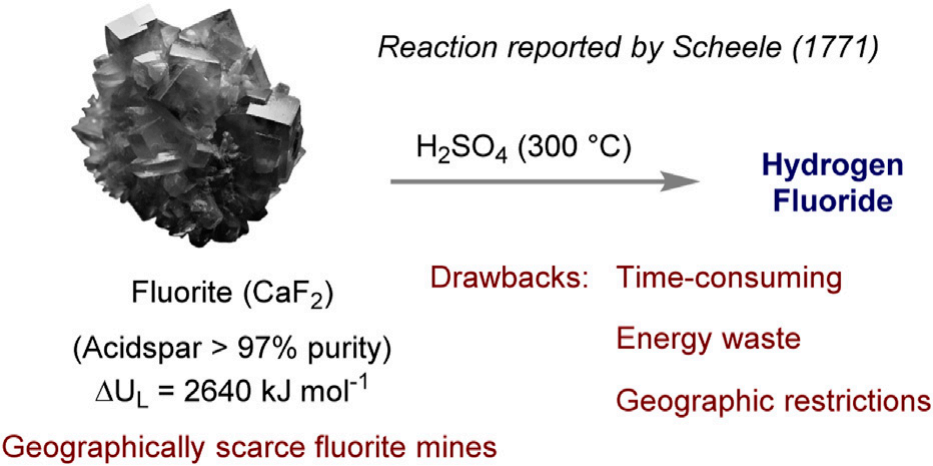
Production methods for AHF between acids and fluorite ore.

Fluorite, also known as fluorspar, is a colorful mineral composed of calcium and fluorine (CaF_2_) (Fuge, 2019). It is commonly found in various geological environments, such as hydrothermal veins, sedimentary rocks, and as a gangue mineral in ore deposits (Zhang et al., 2023). Fluorite has diverse uses and is utilized as a flux in glass and enamel manufacturing, as well as a source of fluorine for the production of fluorine-containing compounds (Fuge, 2019). Given its unique properties and wide range of applications, fluorite plays a crucial role as a mineral resource in various industries (Chang et al., 2022). Among them, the reaction of fluorite ore and acids is one of the most important methods for the production of anhydrous hydrogen fluoride (AHF) (Figure 2) (Patel et al., 2023). The process begins by combining concentrated sulfuric acid (H_2_SO_4_) with fluorspar, resulting in the formation of hydrogen fluoride and calcium sulfate. The hydrogen fluoride is then distilled to obtain AHF once it has been converted into gas. Nonetheless, this conventional approach presents several drawbacks: it is time-consuming, dependent on costly materials, and produces harmful gases that necessitate meticulous management to prevent environmental damage. Furthermore, the dependency on geographically scarce fluorite mines constrains AHF production and impedes the expansion of related industries. The purification of AHF in this method is also laborious, requiring multiple distillation rounds and occasionally recrystallization to remove impurities. While these traditional techniques have played a significant role in supporting various sectors, they are burdened by high costs and environmental concerns. Importantly, fluorite is an unsustainable mineral resource, and the sustainability of fluorite as a mineral resource raises concerns about its long-term availability and its impact on industries that rely on it (Gao et al., 2021). The extraction of fluorite can also have adverse environmental effects, including habitat destruction, soil erosion, and water pollution. Moreover, refining fluorite often involves the use of chemicals and energy-intensive processes, contributing to greenhouse gas emissions and further environmental degradation (Huang et al., 2022).

To address these challenges, new methodologies are being explored, particularly those that utilize readily available industrial by-products. These approaches show promise for both research and practical applications in the industry. By adopting such methods, it is possible to overcome the limitations associated with conventional techniques, including high costs and environmental concerns. This shift towards innovative processes not only enhances the sustainability of AHF production but also mitigates the environmental impacts associated with fluorite extraction and refining.

### 1.2 Introduction of fluorosilicic acid

The phosphate fertilizer industry is crucial for modern agriculture as it provides the essential nutrients required to enhance crop growth and food production (Joshi, 2022). However, the process of extracting phosphorus from phosphate rock generates several byproducts, including fluorosilicic acid (H_2_SiF_6_) (Dahlke et al., 2016). This acid results from the reaction of fluoride-containing minerals with sulfuric acid, which is used to liberate phosphoric acid from raw phosphate ore (Joshi, 2022). The multifaceted environmental hazards of fluorosilicic acid include its pronounced toxicity to aquatic life. When released into water bodies, either through direct discharge or leaching from waste piles, fluorosilicic acid can cause a rapid decline in pH. This increase in acidity can be detrimental to fish and other aquatic organisms, disrupting reproductive cycles, affecting growth rates, and, in severe cases, leading to mass die-offs. Additionally, the fluoride component can bioaccumulate in aquatic organisms, potentially to toxic levels. This biomagnification can have far-reaching consequences, extending up the food chain to predatory species, including humans. The silicon component, though less toxic, can contribute to water turbidity and sedimentation issues, affecting the clarity and quality of water, which in turn impacts photosynthetic aquatic plants and the organisms that depend on them. The release of fluorosilicic acid into soil systems presents another environmental challenge. Fluoride ions can bind to soil particles, making them less available to plants. However, over time, these ions can be released back into the soil solution, where they can be absorbed by plant roots, leading to fluorosis in plants characterized by leaf-tip burn, reduced growth, and, in extreme cases, plant death. The silicon in fluorosilicic acid can also alter the physical structure of the soil, potentially affecting its aeration and water-holding capacity.

The disposal of fluorosilicic acid is another area of concern. Traditionally, the substance has been neutralized with lime and then either stored in large waste ponds or sold as a water fluoridation agent. However, the storage option carries the risk of pond leakage, potentially contaminating groundwater resources. The use of fluorosilicic acid for water fluoridation, although seen as a beneficial recycling method, is contentious due to the potential for overexposure to fluoride and associated health risks. A promising solution is the conversion of fluorosilicic acid into useful materials. Researchers have been investigating ways to transform this byproduct into AHF production. Such applications not only provide a means of recycling but also add economic value, incentivizing the reduction of environmental release.

### 1.3 Fluorosilicic acid as a potential source for AHF production

**SCHEME 1:**
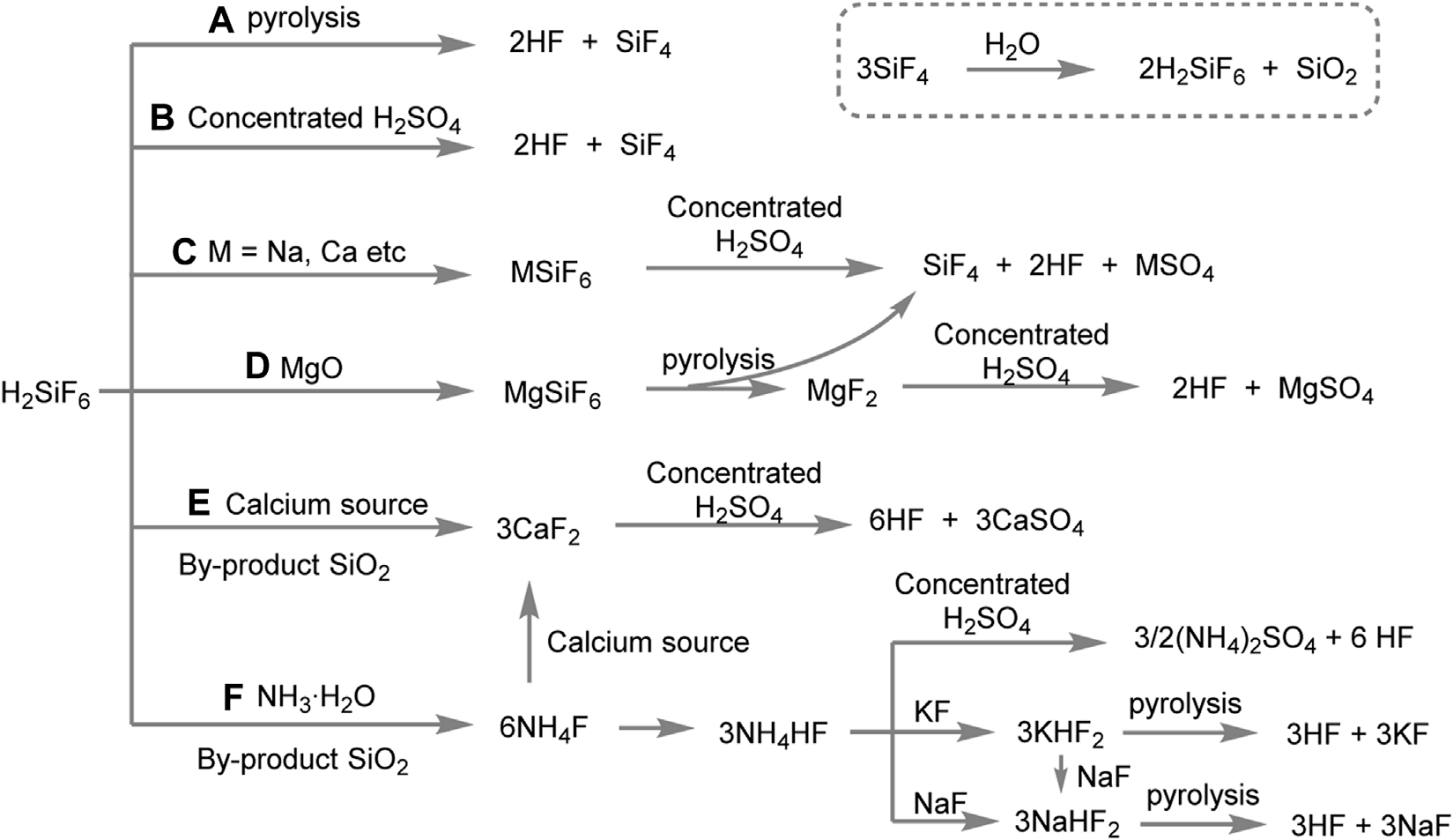
Production of anhydrous hydrogen fluoride from fluorosilicic acid.

Fluorosilicic acid (H_2_SiF_6_) has emerged as a potentially significant source for the production of anhydrous hydrogen fluoride (AHF) in the ongoing search for sustainable, efficient, and economically viable chemical production (Kounbach et al., 2022; Yang et al., 2022). Its potential application in AHF synthesis has caused a shift in perspective, as it offers not only a rich source but also an environmentally friendly method of disposal (Górecki, 1994). The process of preparing hydrofluoric acid from fluosilicic acid can be divided into two methods: the direct method and the indirect method. The direct method involves the direct thermal decomposition of fluosilicic acid (Scheme 1A) or its decomposition using concentrated sulfuric acid to produce hydrofluoric acid (Scheme 1B). In contrast, the indirect method does not directly utilize fluosilicic acid for preparing hydrofluoric acid. Instead, it entails the conversion of fluosilicic acid into various fluorides, such as calcium fluoride, calcium fluosilicate, sodium fluosilicate, magnesium fluosilicate, potassium/sodium bifluoride, and ammonium fluoride. These fluorides are subsequently subjected to thermal decomposition or decomposition with concentrated sulfuric acid to obtain hydrofluoric acid (Schemes 1C–F).

This review aims to analyze the production of AHF from fluorosilicic acid, examining relevant research studies and outlining the methods, mechanisms, and efficiencies of the reactions involved. Additionally, a comparative analysis of AHF synthesis from fluorosilicic acid versus traditional methods will be conducted to quantitatively assess the environmental impact of each approach and determine the most sustainable option. The objective of this review is to stimulate further research into AHF production from fluorosilicic acid by identifying potential challenges and areas for improvement, ultimately advancing this method toward large-scale industrial implementation.

## 2 Methods for the production of AHF from fluorosilicic acid

The process of producing hydrofluoric acid from fluorosilicic acid, a by-product of phosphate ore processing, is focused on the efficient separation of fluorine and silicon, as well as the effective recovery of hydrofluoric acid. This technology is of great significance as it enables the utilization of fluorosilicic acid, which was previously discarded as waste, to generate valuable hydrofluoric acid and other useful products. Moreover, this process contributes immensely to resource efficiency and environmental sustainability in the phosphate ore processing industry.

### 2.1 Direct methods

#### 2.1.1 Direct pyrolysis of fluorosilicic acid

**SCHEME 2:**
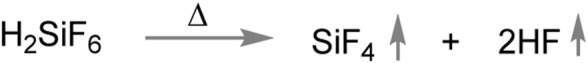
Direct pyrolysis of fluorosilicic acid.

The BUSS ChemTech AG (BCT) has developed a technology for the thermal decomposition of fluorosilicic acid (Dahlke et al., 2016). This process involves the direct decomposition of fluorosilicic acid into hydrogen fluoride (HF) and silicon tetrafluoride (SiF_4_) at a temperature of 150°C. The chemical reaction equation for this process is shown in Scheme 2 as below:

In this process, polyethers and polyethylene glycols are employed as solvents for the absorption of hydrogen fluoride, whereas water is utilized for the absorption of tetrafluorosilane gas, which yields recycled fluorosilicic acid as a raw material. The organic absorbent is subsequently separated by means of fractional distillation, resulting in the production of hydrogen fluoride. This process exhibits several advantages, including a concise procedure, a reduced range of raw materials, and the capability to recycle organic solvents within the system. However, it entails certain technical complexities, such as the requirement to evaporate a substantial volume of water, leading to high energy consumption. Furthermore, stringent control of process conditions and adherence to stringent equipment material specifications pose significant challenges.

Another method involves heating fluorosilicic acid to decompose it into silicon dioxide and dilute hydrogen fluoride (Reed, 1997). Anhydrous hydrogen fluoride can be produced through the treatment with sulfuric acid. However, this technology has the drawback of generating HF with limited purity and requiring a large amount of concentrated sulfuric acid. Mani et al. employed electrodialysis to purify the mixed solution obtained from the thermal decomposition of the fluosilicic acid solution (Mani and Chland, 1983). Nevertheless, this method is associated with disadvantages including complex processing, relatively low technological maturity, and high energy consumption.

#### 2.1.2 Decomposition of fluorosilicic acid by sulfuric acid

**SCHEME 3:**
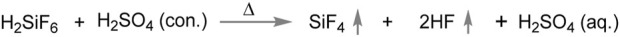
Decomposition of fluorosilicic acid by sulfuric acid.

The United States Wellman Power Gas (Wellman Power-Gas) company developed a process of sulfuric acid decomposition of fluorosilicic acid to obtain hydrofluoric acid (Li et al., 2010). Fluorosilicic acid is dehydrated and pretreated with concentrated sulfuric acid at 125°C. The solution reacts with concentrated sulfuric acid to produce hydrogen fluoride (Scheme 3). The decomposed hydrogen fluoride is absorbed into fluorosulfonic acid by sulfuric acid. The escaped SiF_4_ gas is absorbed and treated with a dilute fluorosilicic acid solution and returned to use. The process corresponds to the chemical reaction depicted in Scheme 3, as illustrated below:

The process route is short, the operation steps are simple, the requirement of the device is low, the added value of the product is high, and it is conducive to improving the overall economic benefit. A notable drawback of the process is the substantial consumption of concentrated sulfuric acid, resulting in the generation of a significant quantity of dilute sulfuric acid containing approximately 70% concentration and carrying a high concentration of fluorine ions. This leads to a considerable loss of fluorine in the system. Consequently, it becomes necessary to appropriately treat the produced sulfuric acid, inevitably resulting in an increase in production costs.

**FIGURE 3:**
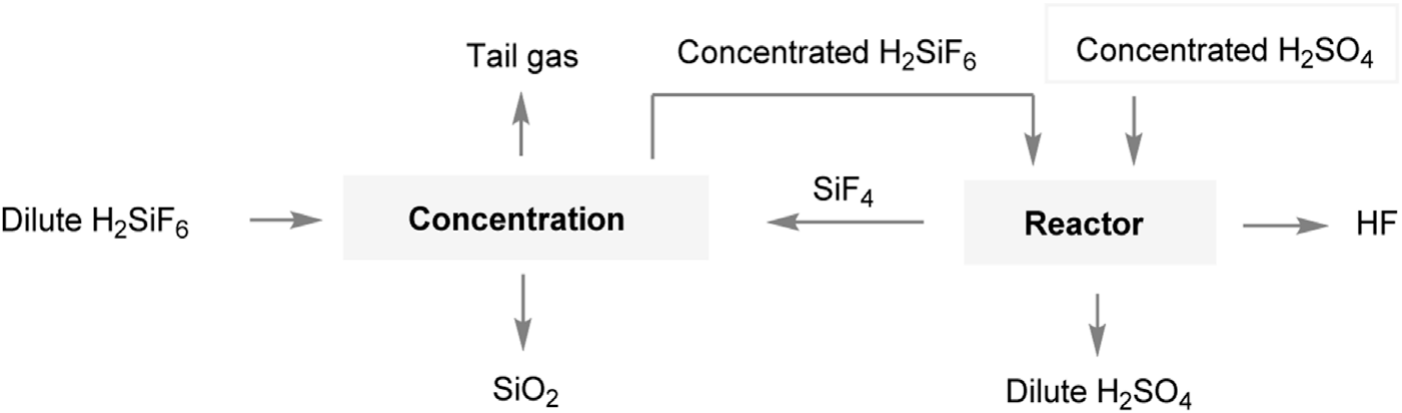
The preparation of AHF by fluorosilicic acid-sulfuric acid method.

Oakley and Mohr et al. made significant advancements in improving the process flow by implementing a circulating system for the silicon tetrafluoride solution. By circulating the solution to the fluosilicic acid concentration step, they were able to efficiently produce fluosilicic acid (Figure 3) (Oakley and Houston, 1962; Mohr et al., 1996). However, one challenge encountered in this process flow is the hydrolysis of silicon tetrafluoride during the generation of fluorosilicic acid. This hydrolysis reaction can lead to the formation of a significant amount of silica gel. The presence of silica gel can complicate the filtration step, making it difficult to carry out. Filtration becomes challenging as the silica gel tends to form colloidal particles, which have a tendency to clog or block filters. As a result, the filtration process may become slower or less effective.

**FIGURE 4:**
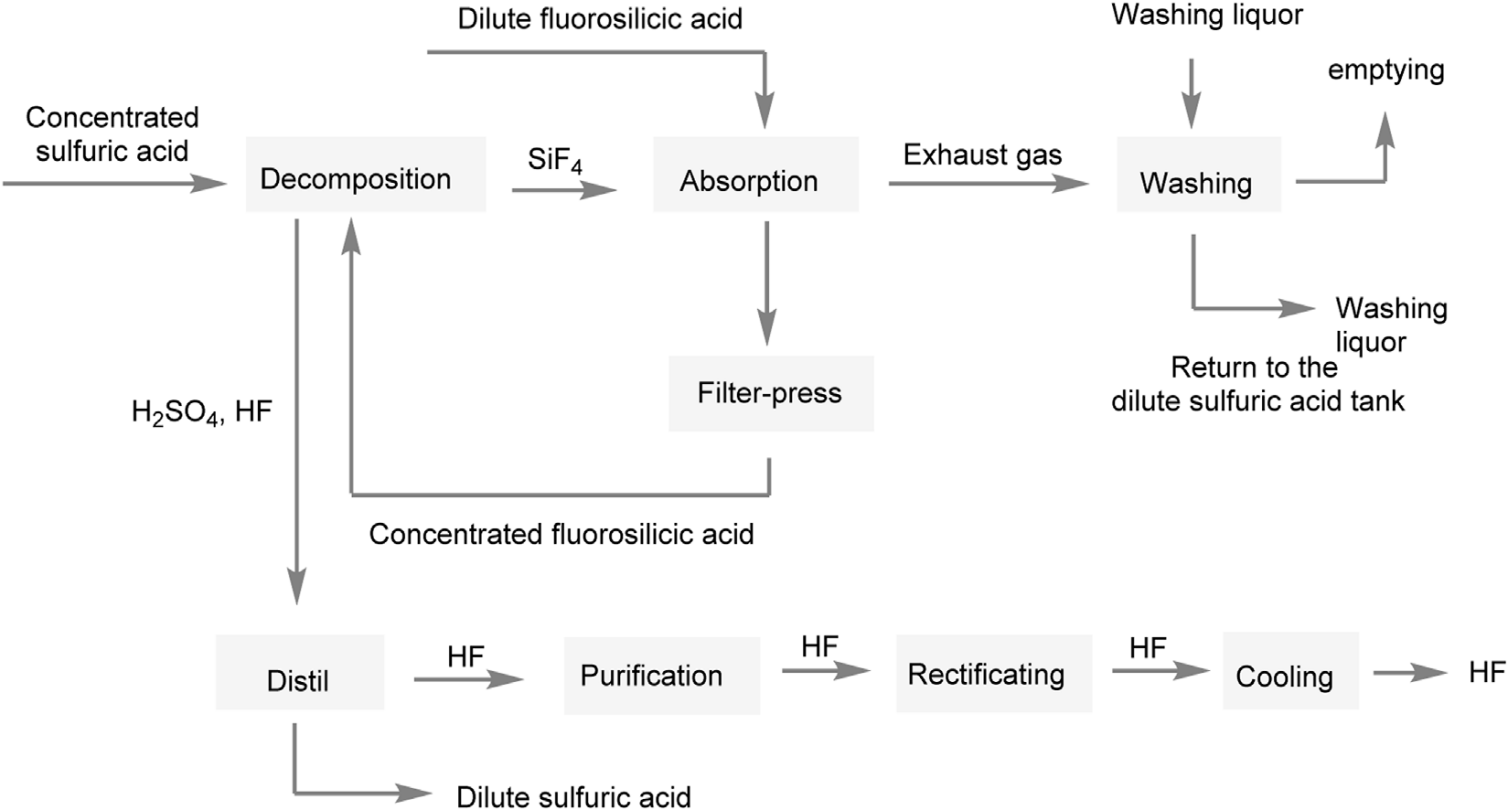
BUSS process.

The BUSS ChemTech AG (BCT) has also conducted systematic research on the process of direct decomposition of fluosilicic acid by concentrated sulfuric acid, forming a relatively mature BUSS process that has been implemented in industry (Figure 4) (Li et al., 2010). However, the byproduct SiO_2_ has not been effectively utilized.

**FIGURE 5:**
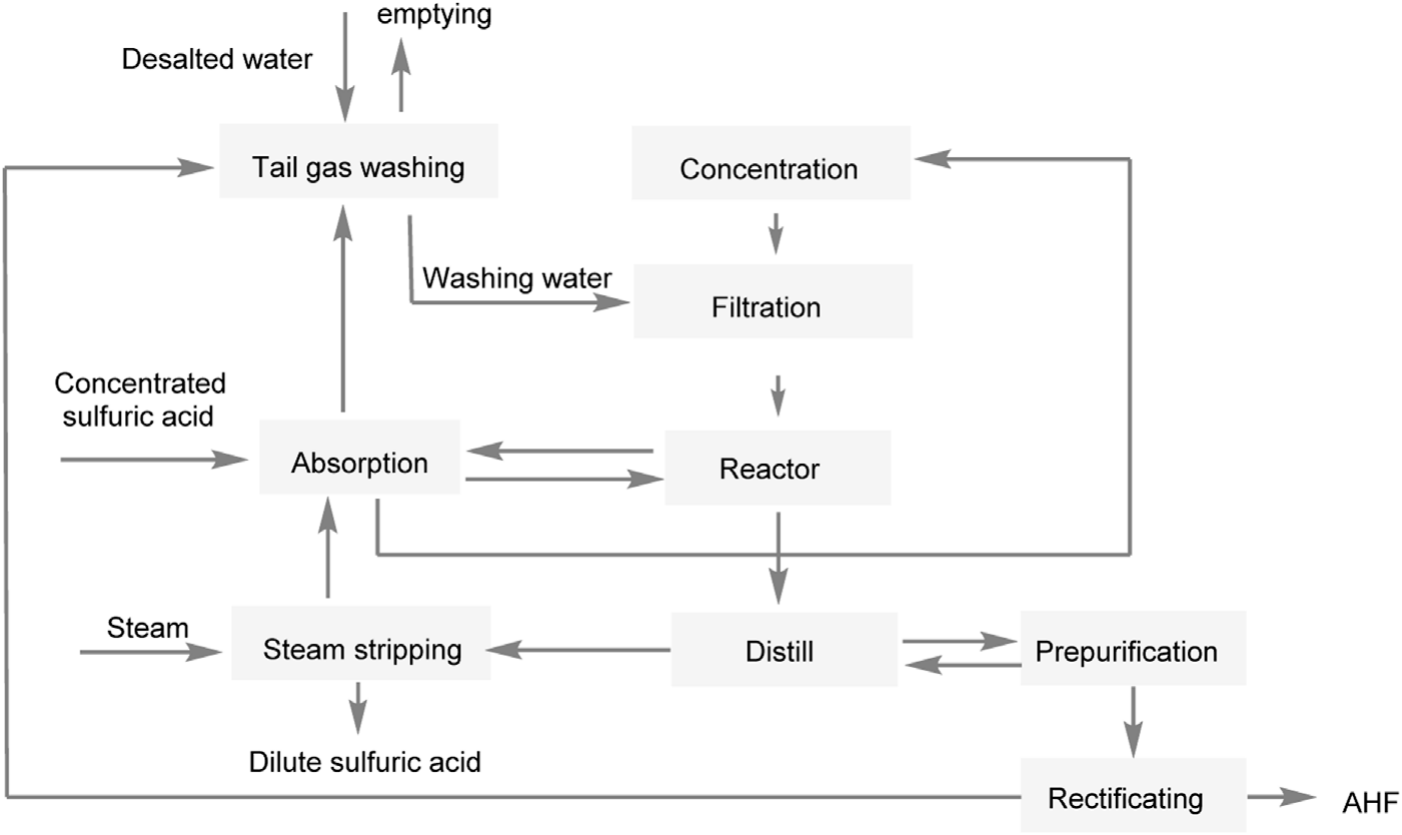
Production of anhydrous hydrofluoric acid in Wengfu Co., Ltd.

Kvaemer AG, a Swiss company, has carried out research on a similar production process and has constructed a pilot production facility. Wengfu Co., Ltd. has further enhanced the efficiency of the hydrogen fluoride production process by modifying its design based on this technology (Tang et al., 2015). In this process, as shown in Figure 5, the diluted fluorosilicic acid obtained from the phosphate fertilizer plant is initially introduced into the concentration system for further concentration. After filtration and separation, the concentrated fluorosilicic acid is then reacted with concentrated sulfuric acid, resulting in the production of silicon tetrafluoride, hydrogen fluoride, and other mixed gases. The mixed gases undergo sulfuric acid absorption, whereby the hydrogen fluoride gas is absorbed and retained by the concentrated sulfuric acid, while the remaining silicon tetrafluoride gas is recycled back into the concentration system for further use. The concentrated sulfuric acid, which has captured the hydrogen fluoride gas, can be subjected to distillation in order to separate the hydrogen fluoride gas. This hydrogen fluoride gas then undergoes purification, and distillation to eliminate high- and low-boiling impurities, and other processes to obtain anhydrous hydrogen fluoride. The diluted sulfuric acid that remains can be returned to the phosphoric acid reactor to be used for phosphoric acid production. This method offers a relatively simple process. However, in theory, the one-way conversion rate of fluorosilicic acid decomposition into hydrogen fluoride is only 33.3%.

### 2.2 Indirect methods

#### 2.2.1 Fluorosilicic acid forms fluorine-containing precipitates with metal cations

When fluorosilicic acid (H_2_SiF_6_) reacts with metal cations, such as calcium (Ca^2+^) or sodium (Na^+^), it forms fluorine-containing salts like sodium hexafluorosilicate (Na_2_SiF_6_) or calcium hexafluorosilicate (CaSiF_6_). These salts are commonly used in various industries for different purposes. To produce hydrogen fluoride (HF), the fluorine-containing salt precipitate is further reacted with concentrated sulfuric acid (H_2_SO_4_) at a specific temperature. This reaction is commonly known as the “fluorosilicic acid process” or “hydrofluoric acid process.”

##### 2.2.1.1 Calcium salts

**SCHEME 4:**
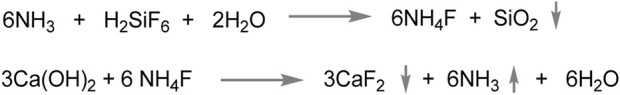
Preparation of CaF_2_ with ammonium fluoride as precipitator.

The US Bureau of Mines employed ammonia (NH_3_) to ammoniate hydrogen hexafluorosilicate (H_2_SiF_6_), resulting in the formation of ammonium fluoride (NH_4_F) and silicon dioxide (SiO_2_) (Scheme 4) (Li et al., 2010). The pH level of approximately 9 was maintained during this reaction. After filtration, calcium hydroxide (Ca(OH)_2_) was added to the filtrate as a precipitating agent to facilitate the production of calcium fluoride (CaF_2_) through the reaction with NH_4_F. The resulting CaF_2_ products were separated and dried. Subsequently, the generated calcium fluoride was used in the conventional fluorspar process to produce HF. It is worth noting that the NH_3_ produced during the precipitation process can be recycled for further use. This process achieves a high total fluorine recovery rate of 97.3% with an ammonia recovery rate of 88.8%. Moreover, it does not require modifications to the HF production equipment. However, it should be acknowledged that the process is characterized by a longer duration and increased complexity.

**SCHEME 5:**
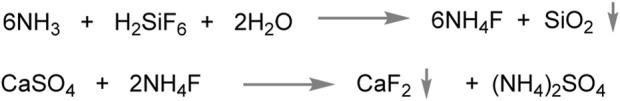
Preparation o\f CaF_2_ with fluorogypsum powder as precipitator.

Yunnan Yuntianhua International Chemical Co., Ltd. developed a comprehensive utilization method for fluorogypsum and fluoride-silicic acid as byproducts in phosphate fertilizer production (Scheme 5) (Li et al., 2010). The method involves the ammoniation reaction of fluorosilicic acid with ammonia water, followed by filtration to remove solid precipitates (white carbon black), resulting in a 5%–22% ammonium fluoride solution. This solution is then mixed and reacted with fluorogypsum powder at temperatures of 60°C–80°C, yielding calcium fluoride with a purity greater than 92% and an ammonium sulfate mother liquor. The calcium fluoride is further reacted with concentrated sulfuric acid using the fluorspar method to obtain hydrogen fluoride products that meet national standards, with the byproduct of reusing the fluorogypsum. Throughout the development process, the reaction conditions are mild, with a high fluoride recovery rate and easily controllable operating conditions. Additionally, the process allows for the production of carbon black and agricultural ammonium sulfate products. However, the process flow is relatively lengthy.

**SCHEME 6:**
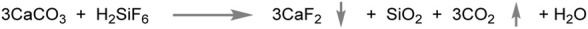
Preparation of CaF_2_ with CaCO_3_ as precipitator.

Various other routes and processes have been explored by different researchers and companies. For instance, Bayer/Kalichemie adopted a methodology involving the reaction of calcium carbonate (CaCO_3_) with H_2_SiF_6_ to produce CaF_2_ and SiO_2_, which were subsequently separated based on density differences (Scheme 6) (Li et al., 2010). Xue et al. also investigated a similar process route, with a molar ratio of hydrogen hexafluorosilicate to limestone set at 1:3. The reaction took place at temperatures between 70°C and 80°C for 2 h. Under these conditions, the reaction rate of limestone reached 93%, with a yield of calcium fluoride surpassing 95% (Li et al., 2010).

The Shandong Lubei Enterprise Group Corporation utilizes sodium fluorosilicate as a raw material for co-production of anhydrous hydrogen fluoride and zeolite molecular sieve (Lv et al., 2021). The process involves the reaction of sodium fluorosilicate with sodium hydroxide solution at temperatures ranging from 60°C to 90°C, resulting in solid-liquid separation and the formation of sodium fluoride solid and sodium silicate solution. Subsequently, sodium fluoride reacts with lime slurry at temperatures between 60°C and 95°C to yield calcium fluoride with a main content exceeding 95%. The production of anhydrous hydrogen fluoride follows the traditional fluorspar process. Furthermore, the sodium silicate solution undergoes sequential mixing, pulping, and crystallization with sodium aluminate solution to obtain zeolite molecular sieve products. This innovative approach not only facilitates the efficient separation and preparation of anhydrous hydrogen fluoride from sodium fluorosilicate but also maximizes the utilization of separated silicon and sodium to generate value-added zeolite molecular sieve products. The process is characterized by a lengthy workflow, with the main challenges lying in the separation of sodium fluoride and sodium silicate, as well as the efficient conversion and separation of sodium fluoride and calcium fluoride.

**FIGURE 6:**
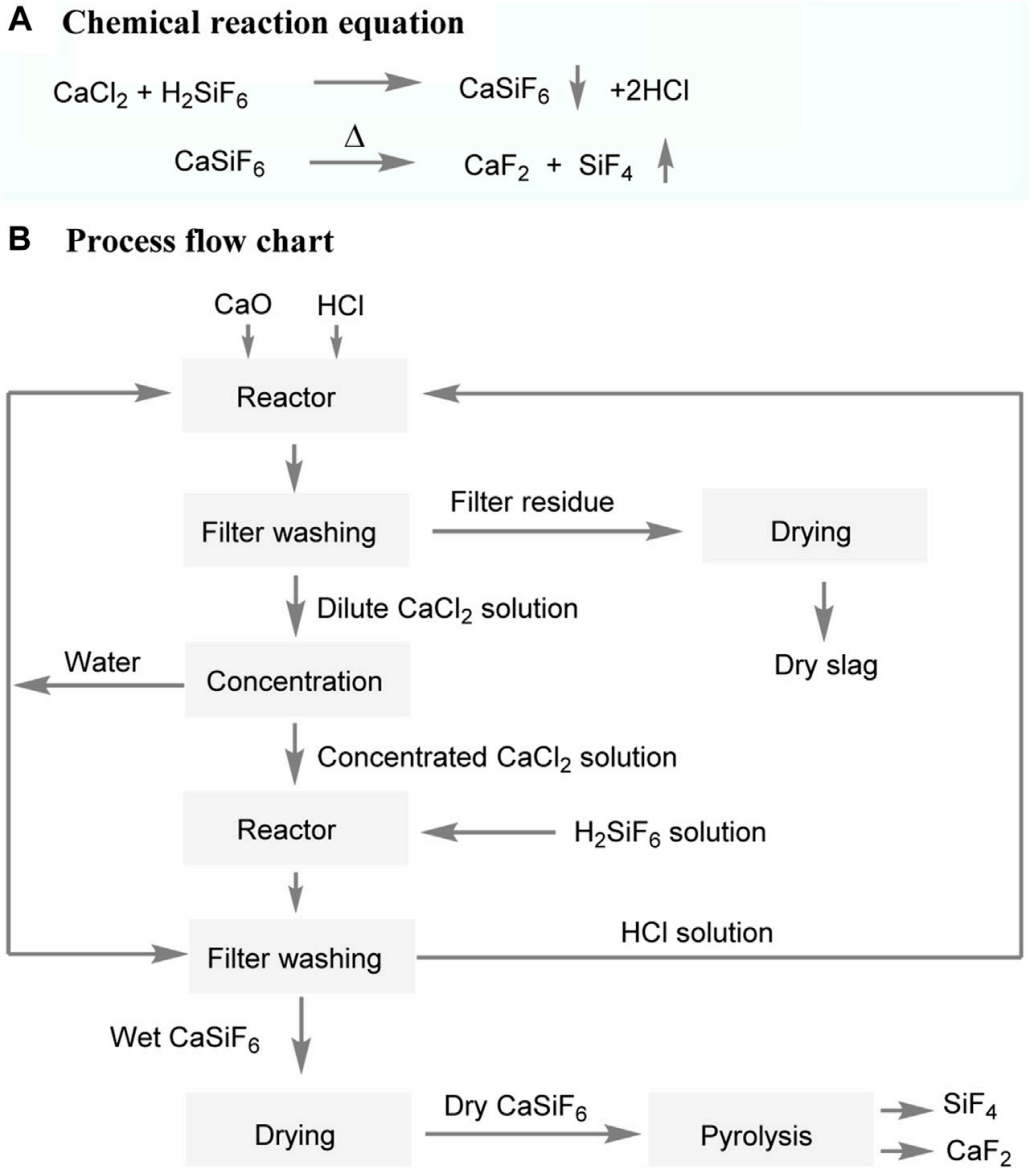
Preparing of CaF_2_ from H_2_SiF_6_ by reaction with CaCl_2_.

In addition to the above, Picardie Aluminium Company in France successfully produced calcium fluosilicate by reacting anhydrous calcium chloride with impure hydrogen hexafluorosilicate (Figure 6) (Li et al., 2010). This process allowed for the precise precipitation of dihydrate calcium fluosilicate under low-temperature conditions by adjusting the concentration of hydrogen hexafluorosilicate and the molar ratio of CaCl_2_ to H_2_SiF_6_. The resulting dihydrate compound was then obtained through filtration, washing, and drying procedures to obtain anhydrous calcium fluosilicate. Calcium fluosilicate can readily decompose into calcium fluoride and silicon tetrafluoride at high temperatures, with the calcium fluoride being suitable for the production of hydrofluoric acid. A yield of anhydrous calcium fluosilicate exceeding 94% is achievable under conditions where the mass concentration of H_2_SiF_6_ exceeds 25% and the molar concentration ratio of CaCl_2_ to H_2_SiF_6_ ranges from 2 to 5. By concentrating a dilute CaCl_2_ solution and reacting it with a hydrogen hexafluorosilicate solution, CaSiF_6_ can be obtained. Further thermal decomposition of CaSiF_6_ within a temperature range of 300°C–400°C results in the formation of CaF_2_. The main challenges of this technology lie in the preparation of calcium fluosilicate, primarily in the filtration of calcium fluosilicate, the selection of calcium sources, and the yield of calcium fluosilicate. Calcium fluosilicate can be completely decomposed at 400°C for 1 h, with the resulting products containing CaF_2_ ≥ 96.5% and SiF_4_ ≥ 87%. The process flow is excessively lengthy, making it difficult to filter calcium fluorosilicate. Furthermore, there are challenges associated with the reuse of wastewater and waste residue.

##### 2.2.1.2 Magnesium salts

Hao (2019) reported a process route for the precipitation of fluosilicic acid using lightly burnt magnesium oxide. The fluosilicic acid magnesium solution was concentrated and dried to obtain solid fluosilicic acid magnesium. Subsequently, the solid was calcined to produce MgF_2_ and SiF_4_ gases. The SiF_4_ gas, after absorption with water, can be continuously recycled. Similar to the fluorspar method, magnesium fluoride is mixed with concentrated sulfuric acid to produce hydrogen fluoride gas and magnesium sulfate. The hydrogen fluoride gas can be refined to obtain anhydrous hydrogen fluoride, while the purified magnesium sulfate can be sold as a byproduct.

**FIGURE 7:**
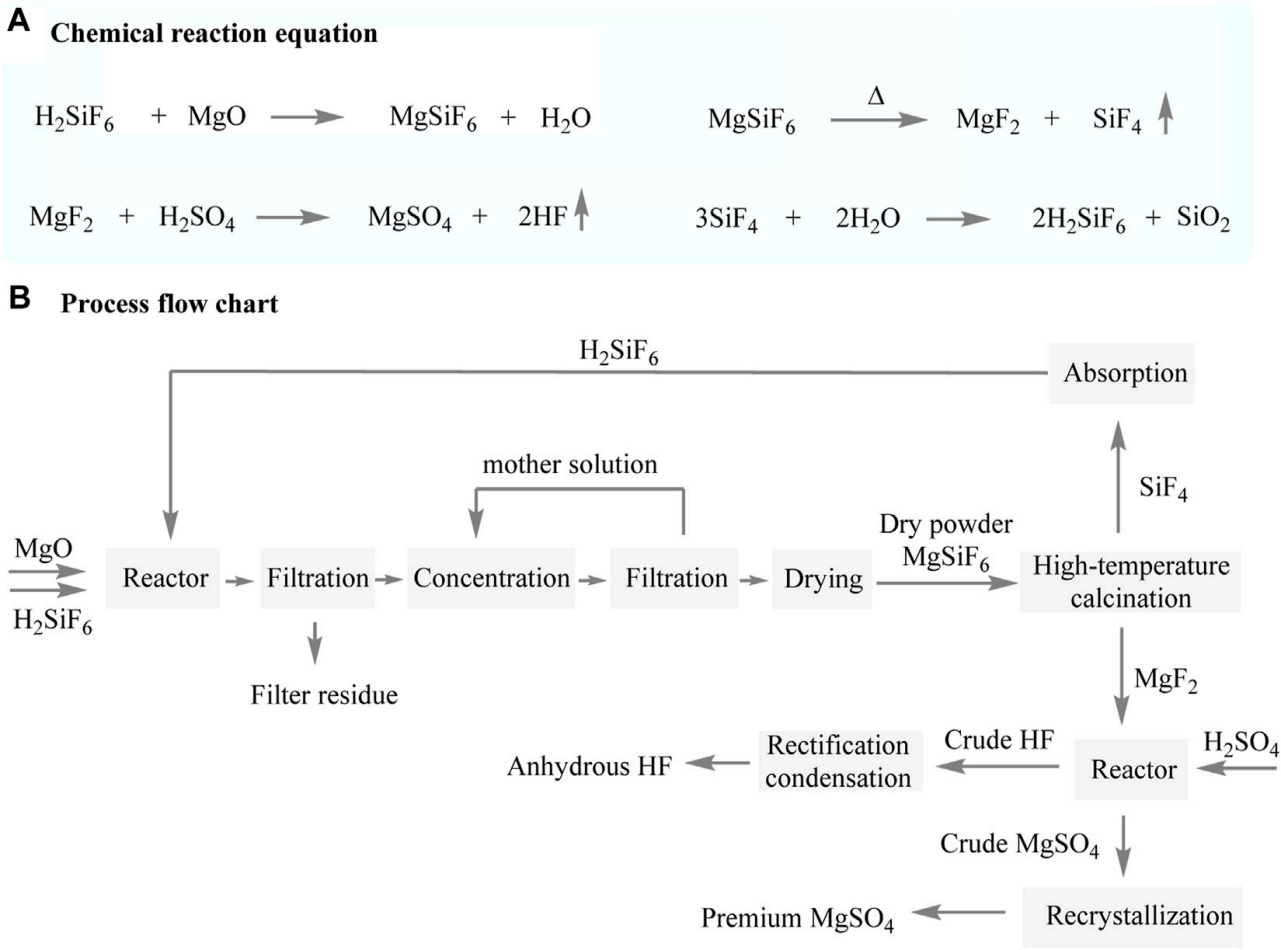
Preparation of AHF by fluosilicic acid and magnesium oxide.

Do-Fluoride New Materials Co., Ltd employs a multi-step process to produce hydrogen fluoride (Figure 7) (Zhang, 2023). In the first step, magnesium oxide reacts with fluorosilicate solution, resulting in the formation of a magnesium fluorosilicate solution. Subsequently, impurities are separated from the solution through filtration to obtain a purified magnesium fluorosilicate solution. This purified solution is then concentrated and subjected to crystallization, leading to the production of magnesium fluorosilicate hexahydrate. The obtained magnesium fluorosilicate hexahydrate undergoes further processing, wherein it is dried at a temperature ranging between 250°C and 300°C. This drying process causes the hexahydrate to decompose, resulting in the formation of magnesium fluoride. In order to obtain hydrogen fluoride, concentrated sulfuric acid is used to decompose the magnesium fluoride. The production process employed by Do-Fluoride New Materials Co., Ltd guarantees efficient and controlled synthesis of hydrogen fluoride from magnesium oxide and fluorosilicate solution. This process is characterized by low production costs, eliminates the need for an ammonia recovery process, and significantly reduces environmental pollution. The produced hydrogen fluoride exhibits a purity of no less than 99.99%, while the by-product magnesium sulfate demonstrates a purity level of at least 99.65%.

#### 2.2.2 Sodium/potassium fluorohydride process

White carbon black, also known as silica, is a synthetic form of silicon dioxide (SiO_2_) that resembles the natural mineral quartz. It is commonly used as a reinforcing filler in various applications due to its white, powdery appearance and desirable properties. With its high surface area and excellent dispersibility, it serves as an additive in industries such as rubber, plastics, coatings, adhesives, and sealants, improving their mechanical properties like tensile strength, tear resistance, and abrasion resistance. Additionally, white carbon black demonstrates anti-blocking and anti-slip characteristics, making it suitable for films and coatings. Its low refractive index makes it suitable for optical applications like optical fibers, glass, and specialty papers. Moreover, it acts as a stabilizer, safeguarding materials from UV damage caused by sunlight exposure.

**FIGURE 8:**
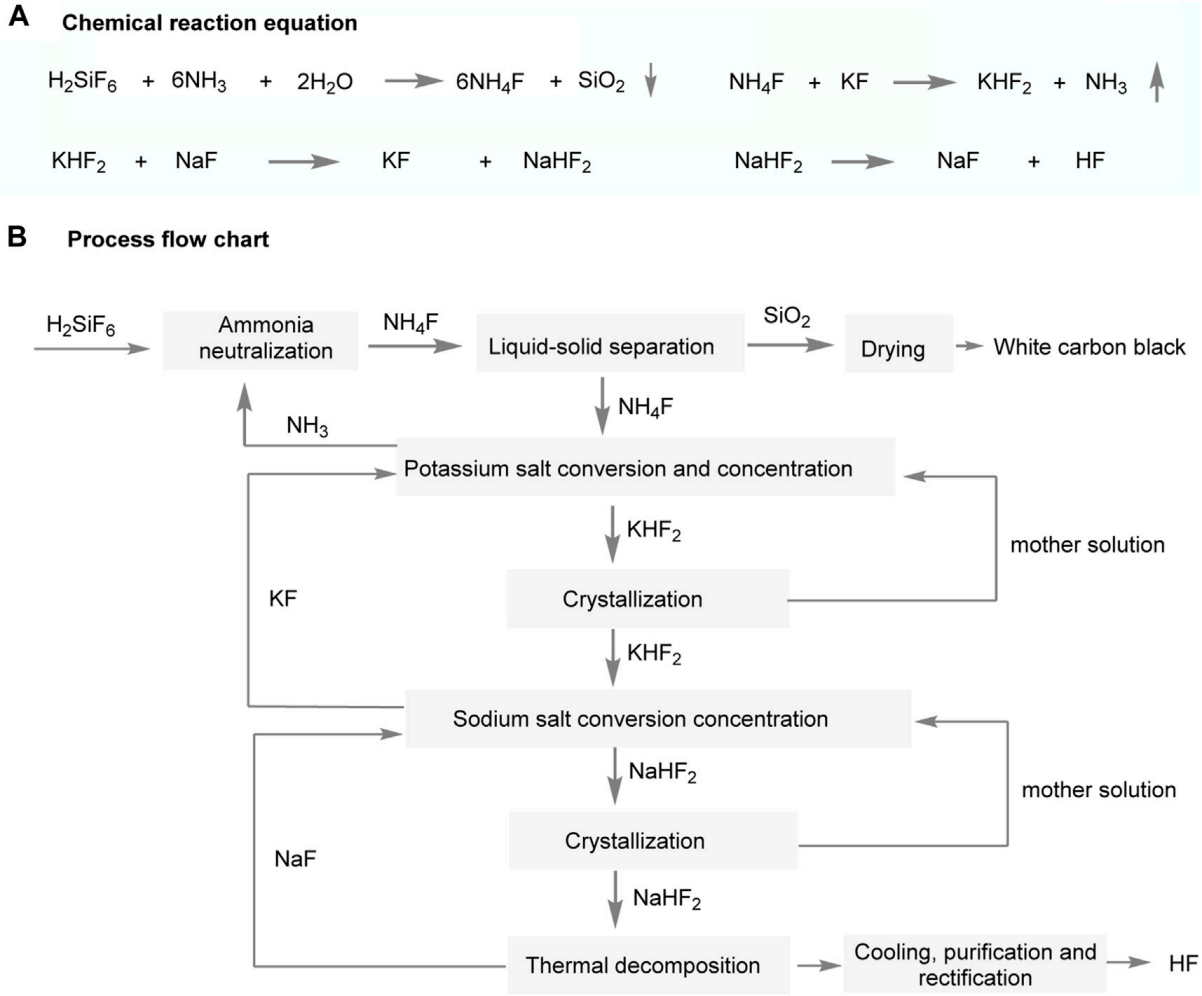
Sodium hydrofluoride process for hydrogen fluoride production.

A collaboration between the British ISC Chemical Company and the Dublin Chemical Company has successfully industrialized a process for producing hydrofluoric acid (HF) and white carbon black from sodium hydrogen fluoride (Figure 8) (Zhang, 2023). The process, illustrated in Figure 6, involves several steps to separate and obtain the desired compounds. Initially, silicofluoride undergoes hydrolysis with ammonia, resulting in the separation of white carbon black and the formation of an ammonium fluoride solution. This solution then reacts with potassium fluoride, yielding potassium hydrofluoride (KHF_2_), which further reacts with NaF to produce sodium hydrogen fluoride (NaHF_2_). The solid NaHF_2_ then undergoes calcination at 300°C, resulting in the decomposition of HF as the primary product and sodium fluoride as a by-product. It is important to note that this technology presents challenges such as high energy consumption, specific material requirements, and difficulties in selecting suitable process equipment. These obstacles must be carefully addressed during implementation.

Researchers and engineers continue to explore ways to enhance the sodium hydrofluoride process, develop better separation techniques, and minimize losses of sodium fluoride and potassium fluoride. These advancements aim to minimize waste, improve overall sustainability, and achieve higher recycling rates in industrial applications. For example, Germany Hanover has made improvements to the sodium hydrofluoride process process by adding potassium fluoride in the same amount as sodium fluoride to produce potassium hydrogen fluoride (Zhang, 2023). This modification allows for the circulation of sodium fluoride and potassium fluoride in the system without any loss. However, achieving 100% recycling of potassium fluoride or sodium fluoride in actual industrialization is difficult. Moreover, controlling the precise amounts of NaF and KF during operations can be a complex task. Industrial environments often have practical limitations that make it challenging to maintain strict control over the quantities of each compound. Another similar process, investigated by Jishou University and the East China Research Institute, follows similar steps but with a key difference (Zhang, 2023). In this process, KHF_2_ is directly thermally decomposed to produce anhydrous HF, eliminating the conversion step involving sodium or potassium salts. Theoretically, this cyclic process using potassium fluoride as a carrier results in no losses. However, this direct calcination process also presents challenges due to the low melting point of potassium hydrogen fluoride, which complicates handling and packaging during high-temperature pyrolysis. Consequently, the overall energy consumption for the entire process is relatively high, and the improvement in economic benefits is not significant.

**FIGURE 9:**
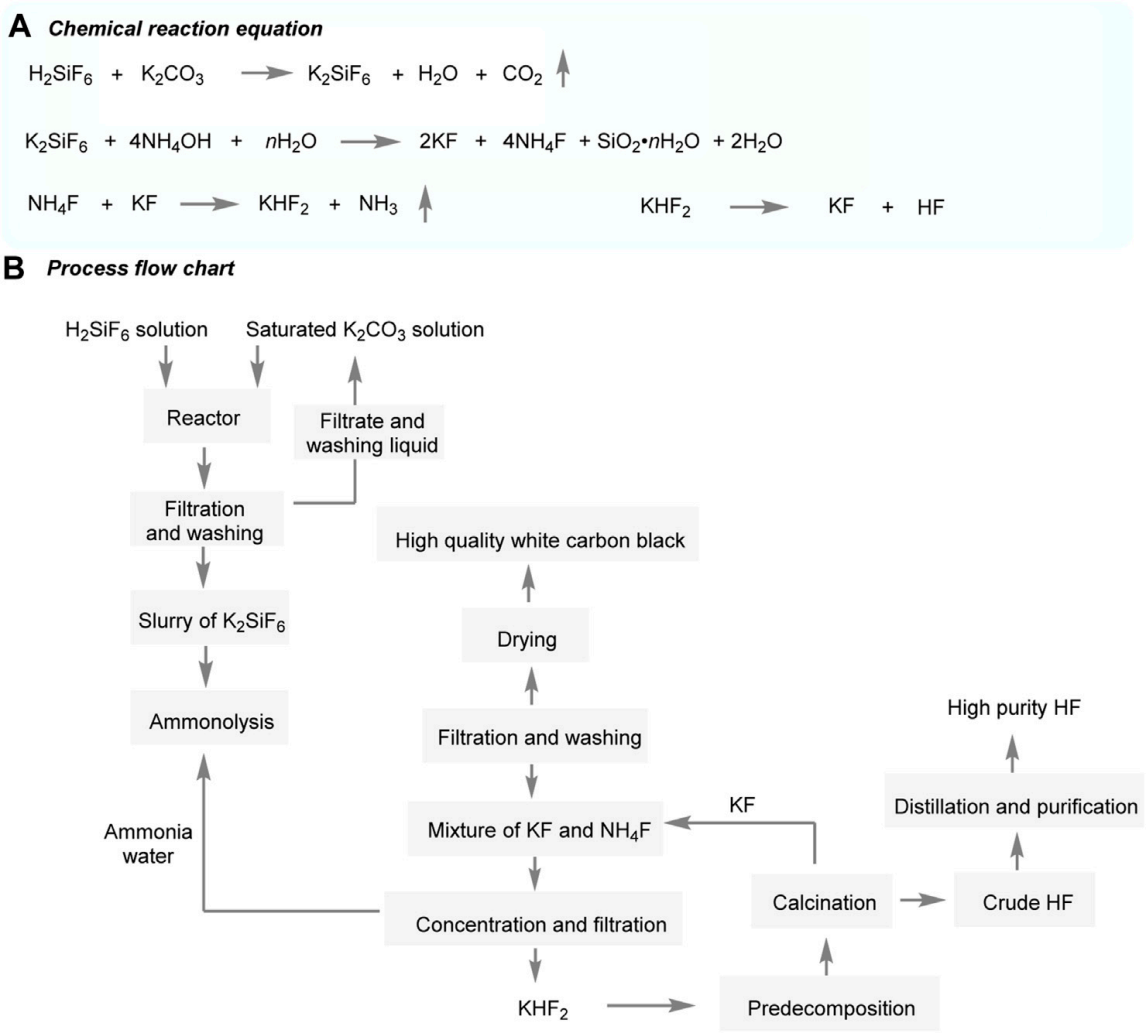
Preparation of AHF from H_2_SiF_6_ using KHF_2_.

Do-Fluoride New Materials Co., Ltd employs a process to neutralize the by-product fluorosilicic acid from phosphate fertilizer (Figure 9) (Cheng et al., 2019). The acid is reacted with a potassium base at a temperature of 90°C, followed by ammoniation within a temperature range of 20°C–40°C until the pH reaches 8 to 8.5. The resulting mixture is filtered to extract white carbon black, and an ammonium fluoride solution is subsequently produced. Then, potassium fluoride is added to the ammonium fluoride solution, followed by concentration, crystallization, and drying at a high temperature to produce potassium hydrogen fluoride. Potassium hydrogen fluoride is predecomposed at a temperature of 150°C–450°C to yield a paste, which is further calcined at 500°C–550°C to obtain crude hydrogen fluoride. The by-product, potassium fluoride, is recycled, and the crude hydrogen fluoride is separated and purified to obtain the final product. The electronic grade hydrofluoric acid produced through this process meets the UPSS standard of the SEMI (Utility Performance Standard for Semiconductor Manufacturing Facilities) standard. The specific surface area of the by-product white carbon black is 205 m^2^/g, making it suitable for use as a rubber reinforcing agent. Moreover, the main content of potassium fluoride in the by-product is ≥ 99.1%, which allows for its exportation. The process involves specific technical aspects, including the concentration of potassium hydrogen fluoride, control of pyrolysis energy consumption, and selection of pyrolysis equipment material. It is important to note that the melting point of potassium hydrogen fluoride is 238.17°C, and its decomposition temperature is higher than its melting point, resulting in a melting phenomenon during the pyrolysis process. This can lead to encasing, sticking to the walls, and caking of the potassium fluoride generated during continuous temperature decomposition, thus reducing the decomposition efficiency. To mitigate this issue, Do-Fluoride New Materials Co., Ltd employs a predecomposition device that first produces a paste, followed by high-temperature calcination to decompose the paste. This alleviates the issue of decreased heat transfer caused by the melting and packaging of potassium hydrogen fluoride, leading to reduced energy consumption.

#### 2.2.3 Ammonium fluoride/ammonium hydrogen fluoride process

**SCHEME 7:**
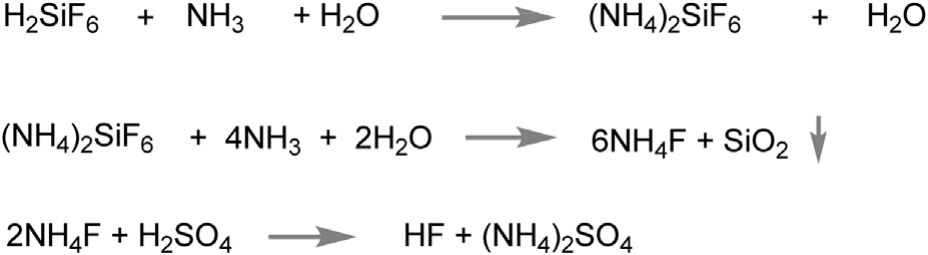
Production of AHF by sulfuric acid decomposes ammonium fluoride.

Guizhou Kailin Group Public and Guizhou Institute of Chemical Engineering have successfully developed an efficient method for obtaining hydrogen fluoride (HF) through the decomposition of ammonium fluoride (NH_4_F). This process, which has been applied in industrialization, offers various practical applications in industries requiring HF as a raw material or reagent (Zhao et al., 2020). As shown in Scheme 7 the process involves several steps. Initially, fluorosilicate acid is ammoniated with ammonia gas, resulting in the formation of solid ammonium fluorosilicate. This solid is then reacted with ammonia water to produce NH_4_F. Finally, concentrated sulfuric acid is used to convert NH_4_F into HF. The successful implementation of this method on a large scale indicates its potential for cost-effective and efficient industrial production. This collaboration highlights the significance of research and development efforts in establishing improved industrial processes.

**FIGURE 10:**
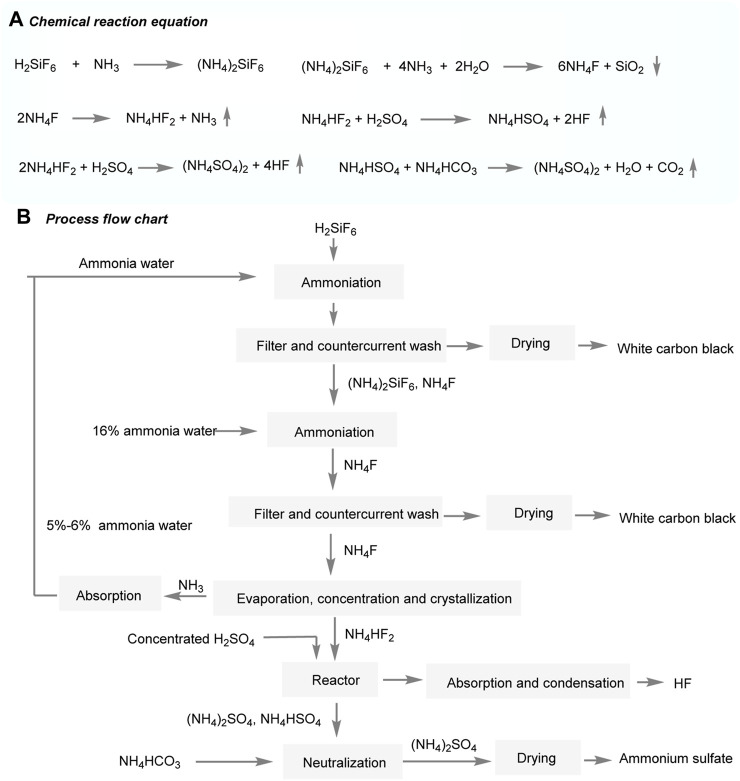
Production of AHF by sulfuric acid decomposition of ammonium hydrogen fluoride.

In a similar vein, the collaboration between Yunnan Yuntianhua International Chemical Co., Ltd. and Tianjin Chemical Design and Research Institute focuses on studying the ammonium hydrogen fluoride process (Figure 10) (Yang et al., 2006). This process involves multiple steps and presents both advantages and challenges. To begin, hydrogen hexafluorosilicate (H_2_SiF_6_) is gently ammoniated at temperatures of 45°C and 35°C, resulting in the formation of a solution containing ammonium fluoride (NH_4_F). This solution is then concentrated and reacts with concentrated sulfuric acid (H_2_SO_4_), yielding hydrogen fluoride (HF) and ammonium sulfate ((NH_4_)_2_SO_4_) (Figure 10). This process can produce white carbon black and ammonium sulfate as by-products. The aggregate morphology and specific surface area of white carbon black can be adjusted by ammoniation conditions. One notable advantage of this process is the ability to recycle ammonia (NH_3_), reducing its consumption as a reagent. However, the process is considered complex due to specific requirements for the production unit. The efficient use of NH_3_ in the actual production process may present challenges, potentially leading to increased by-product formation and higher production costs.

## 3 Conclusion

In conclusion, this review has critically examined the method of producing anhydrous hydrogen fluoride (AHF) from fluorosilicic acid, which is an abundant by-product and industrial waste from the phosphate fertilizer industry. The implications of the reviewed transformation process are far-reaching, demonstrating the possibility of efficient, cost-effective, and more sustainable method of anhydrous hydrogen fluoride (AHF) production. The application of this methodology not only attains resource efficiency but also provides a positive solution to the waste management issue, specifically regarding the abundant by-product waste from the phosphate fertilizer industry, fluorosilicic acid (H_2_SiF_6_).

The potential benefits of the investigated transformation process are numerous. Primarily, implementing the thermal hydrolysis process for AHF synthesis by employing fluorosilicic acid as starting materials facilitates a reduction in reliance on mineral fluorspar. This reduction can lead to significant cost savings and a decrease in environmental degradation caused by mining activities.

Secondly, this process redefines fluorosilicic acid from a waste product to a valuable resource for AHF production. Instead of incurring costs and potential environmental hazards associated with disposing of this industrial waste, it can be utilized in a meaningful manner, contributing to sustainable industrial practices. The additional advantage is the remarkable opportunity it offers for comprehensive industrial symbiosis where waste from one industrial process becomes the feedstock for another, symbolizing an integrated industrial ecosystem.

Finally, this thermal hydrolysis method has shown potential in terms of reaction efficiency, indicating that the process could be industrially applicable. This suggests that the approach can be further scaled up and optimized to fulfill the increasing demand for AHF in various sectors, hence, positively influencing industrial growth while adhering to the principles of green chemistry and sustainability.

Therefore, the implications and potential benefits of the reviewed transformation process mark a significant stride forward for more sustainable and greener AHF production, contributing to the overarching objectives of circular economy and sustainable industrial development. Further explorative and optimizing research in this approach is highly encouraged to harness its full potential.
